# Risk-Adapted Empiric Therapy for Acute Cholangitis: Beyond the Broad- Versus Narrow-Spectrum Dichotomy

**DOI:** 10.7759/cureus.111582

**Published:** 2026-06-26

**Authors:** Sakue Masuda, Yoshinori Imamura, Karen Kimura, Makomo Makazu, Kazuya Koizumi

**Affiliations:** 1 Gastroenterology Medicine Center, Shonan Kamakura General Hospital, Kamakura, JPN; 2 Cancer Care Promotion Center, University of Fukui Hospital, Eiheiji, JPN

**Keywords:** acute cholangitis, antimicrobial resistance, antimicrobial stewardship, biliary drainage, broad-spectrum antibiotics, de-escalation, empiric therapy, risk-adapted therapy

## Abstract

Acute cholangitis requires both timely source control and appropriate empiric antimicrobial therapy. Although unnecessarily broad antimicrobial coverage should be avoided from an antimicrobial stewardship perspective, the choice of empiric therapy should not be reduced to a simple comparison between broad- and narrow-spectrum regimens. Patients with biliary stents, repeated biliary interventions, recurrent cholangitis, healthcare exposure, proton pump inhibitor use, prior resistant-organism isolation, or prior antimicrobial exposure may have an increased risk of antimicrobial-resistant pathogens. In such patients, broad-spectrum empiric therapy may be selected not as routine overtreatment, but as risk-adapted treatment to avoid inadequate initial coverage. Observational studies evaluating empiric antimicrobial spectrum should therefore account for patient-level resistant-pathogen risk, microbiological findings, source-control details, and de-escalation practices. A risk-adapted approach may better balance antimicrobial stewardship with patient safety in acute cholangitis.

## Editorial

Antimicrobial stewardship is increasingly important in the management of acute cholangitis. Because biliary drainage can rapidly control the source of infection in many patients, unnecessarily broad or prolonged antimicrobial therapy should be avoided whenever possible. However, the interpretation of empiric antimicrobial spectrum in acute cholangitis should not be reduced to a simple comparison between broad-spectrum and narrow-spectrum therapy. The more clinically relevant question is whether broad-spectrum empiric therapy can be targeted to patients with a sufficiently high risk of antimicrobial-resistant pathogens.

Recent observational evidence has raised important questions about the role of broad-spectrum empiric therapy in acute cholangitis. A large Japanese claims database study reported that broad-spectrum empiric therapy was not associated with improved mortality compared with narrow-spectrum therapy [1]. In that study, broad-spectrum therapy was operationally defined as the use of carbapenems, piperacillin/tazobactam, or fourth-generation cephalosporins, whereas all other regimens were categorized as narrow-spectrum therapy [1]. Clinically, however, the distinction between broad- and narrow-spectrum empiric therapy depends not only on drug class but also on expected pathogens, local susceptibility patterns, and patient-level risk factors for resistant organisms.

Narrower regimens are generally intended to cover common community-acquired biliary pathogens, including Enterobacterales such as *Escherichia coli* and *Klebsiella *spp., streptococci, and anaerobes when indicated [2]. *Enterococcus *spp. are also important biliary pathogens, particularly in healthcare-associated infection or patients with prior biliary intervention [2]. Broader regimens may be selected when coverage for resistant Gram-negative organisms, *Pseudomonas aeruginosa*, or *Enterococcus *spp. is considered necessary. Existing guidelines, including the Tokyo Guidelines 2018, recommend selecting empiric antimicrobial therapy according to disease severity, community-acquired or healthcare-associated setting, local susceptibility patterns, and the patient’s clinical condition [2]. This guideline-based framework supports antimicrobial stewardship, but it also implies that empiric therapy should be individualized rather than applied uniformly.

In clinical practice, broad-spectrum agents are often selected for patients perceived to be at increased risk for resistant organisms. Relevant risk factors include recent antimicrobial exposure, recent hospitalization, residence in a long-term care facility, prior biliary instrumentation, biliary stents, recurrent cholangitis, prior resistant-organism isolation, healthcare exposure, and proton pump inhibitor use [3-5]. Cumulative antimicrobial exposure and prolonged antimicrobial administration may also contribute to the emergence of resistant organisms [3,5]. Many of these factors are familiar to clinicians managing biliary infections, but they may not be sufficiently represented in administrative databases.

This limitation is important because administrative databases may identify diagnoses, procedures, and prescriptions, but they often lack granular information on prior culture results, colonization with resistant organisms, stent duration and indication, local antibiograms, and the clinician’s rationale for empiric antimicrobial selection. Therefore, if patients in the broad-spectrum group have a higher baseline risk of resistant-pathogen infection, similar mortality between broad-spectrum and narrow-spectrum groups should not automatically be interpreted as evidence that broad-spectrum therapy is unnecessary. Rather, clinicians may have preferentially selected broad-spectrum agents for patients perceived to be at higher risk for resistant pathogens, and such treatment may have prevented worse outcomes that could have occurred with inadequate narrow-spectrum coverage. This is a form of confounding by indication that is difficult to eliminate completely in observational research.

Therefore, the key clinical question is not whether broad-spectrum empiric therapy should be used routinely in acute cholangitis. Routine broad-spectrum therapy may increase unnecessary antimicrobial exposure and contribute to antimicrobial resistance. Conversely, overly restrictive empiric therapy may be harmful in selected patients with a high probability of resistant pathogens and a risk of inadequate initial coverage. A risk-adapted approach is needed to balance antimicrobial stewardship with patient safety.

Risk-adapted empiric therapy requires careful assessment of both disease severity and resistant-pathogen risk. Severity indicators used for adjustment in the claims database study, such as sepsis, intensive care unit admission, and immunosuppression, are important, but they do not fully capture the likelihood of resistant biliary pathogens [1]. Biliary instrumentation history, healthcare exposure, cumulative antimicrobial exposure, and prior microbiological information, among other factors, should also be considered [2-5]. Patient-related factors such as renal or hepatic dysfunction, advanced age, pregnancy, and drug allergies may further influence the choice, dosing, and safety of empiric agents, although they are not necessarily direct markers of resistant-pathogen risk. Once microbiological data become available and adequate source control has been achieved, de-escalation should be pursued whenever feasible.

Future studies evaluating empiric antimicrobial strategies for acute cholangitis should distinguish routine broad-spectrum empiric therapy from risk-adapted broad-spectrum therapy. Such studies should incorporate patient-level resistant-pathogen risk stratification, microbiological findings, source-control details, and de-escalation practices. This approach may provide a more clinically meaningful framework than a simple broad- versus narrow-spectrum comparison.
